# Powassan Encephalitis: A Case Report from New York, USA

**DOI:** 10.1155/2022/8630349

**Published:** 2022-08-17

**Authors:** Danielle A. Bazer, Matthew Orwitz, Nicholas Koroneos, Olga Syritsyna, Elizabeth Wirkowski

**Affiliations:** Department of Neurology, The State University of New York Stony Brook, Renaissance School of Medicine, 101 Nicolls Road, HSC 12/20, Stony Brook, NY 11794, USA

## Abstract

**Background:**

Powassan is a positive-sense, single-stranded, enveloped RNA virus that is a tick-borne *Flavivirus,* transmitted by *Ixodes* species, with groundhogs being the usual mammalian host. The virus is endemic to North America, with peak transmission during the summer and fall. The incubation period is 7–34 days, followed by a prodrome of flu-like symptoms. Although most infected individuals are asymptomatic, the virus can penetrate the CNS to produce a viral encephalitis. The key to the diagnosis is a positive serology.

**Results:**

The patient is a 62-year-old male with a past history of a right putamen infarct, hepatitis C, hypertension, and substance abuse who presented due to acute onset altered mental status, dysarthria, and left-sided facial droop. He had several tick bites around the time of presentation in December. He was empirically treated for possible meningitis, as CSF revealed WBC 370 (80% mononuclear cells); RBC 10, protein 152 mg/dL, and glucose 59 mg/dL. An MRI scan of the brain showed a subacute left putamen stroke. MRAs of the head and neck were unremarkable. A Mayo Clinic Encephalopathy Panel was unremarkable; however, a New York State Arbovirus panel revealed Powassan IgM ELISA as well as Powassan Polyvalent microsphere immunofluorescence assay reactivity. His hospital course was complicated by critical illness myopathy and respiratory failure requiring tracheostomy.

**Conclusion:**

The *Powassan virus* is a known etiology for encephalitis in North America. Although the peak incidence of transmission is in the summer and fall, this does not exclude transmission during other seasons. Due to the increasing prevalence of *Powassan virus* in Lyme-endemic areas particularly in the Midwest and Northeast, United States, patients with an unexplained altered mental status in these regions should be screened for *Powassan virus*, regardless of the time of year.

## 1. Background

Powassan is a positive-sense [1], single-stranded, enveloped RNA virus that is a tick-borne *Flavivirus,* transmitted by *Ixodes* species, with groundhogs and other small- to medium-sized forest rodents being the usual mammalian host [2–4]. This virus is endemic to North America, with peak transmission during the summer and fall, when the *Ixodes* tick is most active [4, 5]. However, as temperatures continue to rise in endemic areas, it is anticipated that the rates of Powassan infection will also rise in other seasons, including the winter [6]. The incubation period is 7–34 days, followed by a prodrome of flu-like symptoms, consistently associated with headache and fever, lasting 1–3 days on average [7]. Despite the virus being highly neurotropic, the majority of patients who are infected with Powassan are asymptomatic [2]. When patients are symptomatic from *Powassan virus*, they will present with encephalitis and altered sensorium [2, 4].

Routine laboratory studies are typically unremarkable; the cerebrospinal fluid will show a pleocytosis, which can be lymphocytic or polymononuclear predominant [2, 8].

The gold standard for diagnosis is serological testing, particularly immunoglobulin M (IgM) antibody testing utilizing enzyme-linked immunosorbent assay (ELISA) and immunofluorescence assay [4]. Although variable, neuroimaging will preferentially show T2-hyperintense lesions without contrast enhancement [2].

There is insufficient data for standardized treatment, as the treatment is largely supportive. However, case reports suggest the usage of intravenous immunoglobulin and steroids [2, 3].

Roughly, 50% of patients will have long term neurological sequelae of *Powassan virus*, such as recurrent headaches, cognitive disruption, and focal neurological deficits [5, 9]. It is estimated that the 10% of patients with Powassan will expire from the disease [5, 9].

## 2. Case Presentation

The patient is a 62-year-old male with a past medical history of a right putamen infarct, hepatitis C, hypertension, and substance abuse who initially presented due to altered mental status, dysarthria, and a left facial droop. He had several tick bites around the time of presentation in December.

**Figure 1:**
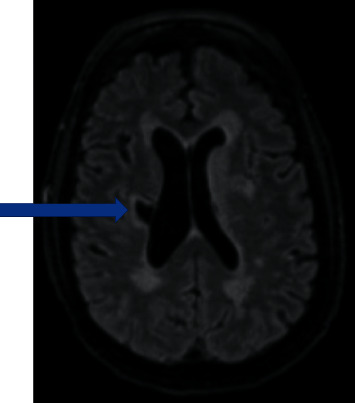
Initial MRI T2 FLAIR delineating chronic right corona radiata and putamen infarcts with ex-vacuo dilation of the right lateral ventricle.

Magnetic resonance imaging (MRI) of the brain showed subtle acute to subacute left putamen infarct (Figure 1). Magnetic resonance angiography (MRA) of the head and neck was unremarkable. Initial lumbar puncture (LP) revealed white blood cells (WBC) 370 (80% mononuclear, reference range: 0–5 cells); red blood cells (RBC) 10 (reference range: 0–2 cells), with elevated protein 152 (reference range: 15–45 mg/dL), glucose 59 (reference range: 40–70 mg/dL). He was empirically treated for possible meningitis with ceftriaxone and acyclovir. He was intubated for airway protection.

**Figure 2:**
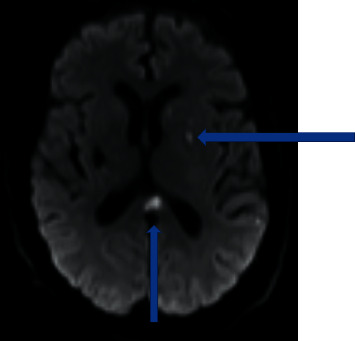
MRI diffusion weighted imaging with acute infarcts in the splenium of the corpus callosum and the posterior superior frontal lobe.

His hospitalization course was complicated by a recurrent stroke; his repeat MRI brain 2 weeks after presentation revealed a small foci of acute infarct involving cerebellum, left basal ganglia, and splenium of corpus callosum (Figure 2). A repeat LP showed WBC 66, RBC 63, protein 87.6, and glucose 68. Electromyography (EMG) and nerve conduction studies (NCS) showed a generalized axon loss with demyelinating polyradiculopathy. He received 2 courses of intravenous immune globulin because of mild demyelinating features on EMG/NCS. A Mayo Clinic Encephalopathy Panel was unremarkable; this panel is used to investigate autoimmune and paraneoplastic etiologies of encephalopathy. A New York State Encephalitis Panel performed with the addition of an *Arbovirus* Panel, given the patient's known tick exposure. This revealed a positive CSF Powassan IgM ELISA as well as Powassan Polyvalent microsphere immunofluorescence assay reactivity, which confirmed the presence of active *Powassan virus* in CSF, a surrogate to diagnose Powassan encephalitis.

Unfortunately, the patient required artificial airway and feeding access due to respiratory failure. At the time of discharge, notable neurological symptoms included global aphasia.

## 3. Discussion

*Powassan virus* is a known etiology for encephalitis in North America. Although the peak incidence of transmission of the virus is in the summer and fall when the *Ixodes* species is most active, this does not exclude transmission in other seasons [5]. As wintertime temperatures continue to rise in endemic areas, it can be anticipated that there will be greater tick activity and exposures in the winter [6]. This suggests that the infection rate will also rise, increasing the number of patients with *Powassan virus*.

Our patient's diagnosis demonstrates the importance of obtaining a thorough tick exposure history. Due to the increasing prevalence of *Powassan* virus in Lyme-endemic areas (particularly in the Midwest and Northeast, United States), some experts have suggested screening for *Powassan virus* in patients with unclear CNS phenomena in Lyme-endemic areas, as the disease is underrecognized [5, 10]. This further highlights the need for increased awareness and surveillance of Powassan infections [6].

## 4. Conclusion

This case highlights the importance of obtaining Powassan serology in a patient with an unexplained altered mental status. It also demonstrates the importance of testing for the virus in the appropriate clinical scenario in Lyme-endemic areas, even outside of the normal tick season.

## Data Availability

The data for this article were gathered from the patient's medical record.
